# Intraoperative cone beam computed tomography for detecting residual stones in percutaneous nephrolithotomy: a feasibility study

**DOI:** 10.1007/s00240-021-01259-1

**Published:** 2021-03-08

**Authors:** R. A. Kingma, M. J. H. Voskamp, B. H. J. Doornweerd, I. J. de Jong, S. Roemeling

**Affiliations:** Department of Urology, University of Groningen, University Medical Center Groningen, House Zip Code CB 62, PO Box 30.001, 9700 RB Groningen, The Netherlands

**Keywords:** Percutaneous nephrolithotomy (PCNL), Cone beam computed tomography (CBCT), Residual fragments, Urolithiasis

## Abstract

Cone beam computed tomography (CBCT) provides multiplanar cross-sectional imaging and three-dimensional reconstructions and can be used intraoperatively in a hybrid operating room. In this study, we investigated the feasibility of using a CBCT-scanner for detecting residual stones during percutaneous nephrolithotomy (PCNL). Intraoperative CBCT-scans were made during PCNL procedures from November 2018 until March 2019 in a university hospital. At the point where the urologist would have otherwise ended the procedure, a CBCT-scan was made to image any residual fragments that could not be detected by either nephroscopy or conventional C-arm fluoroscopy. Residual fragments that were visualized on the CBCT-scan were attempted to be extracted additionally. To evaluate the effect of this additional extraction, each CBCT-scan was compared with a regular follow-up CT-scan that was made 4 weeks postoperatively. A total of 19 procedures were analyzed in this study. The mean duration of performing the CBCT-scan, including preparation and interpretation, was 8 min. Additional stone extraction, if applicable, had a mean duration of 11 min. The mean effective dose per CBCT-scan was 7.25 mSv. Additional extraction of residual fragments as imaged on the CBCT-scan occurred in nine procedures (47%). Of the follow-up CT-scans, 63% showed a stone-free status as compared to 47% of the intraoperative CBCT-scans. We conclude that the use of CBCT for the detection of residual stones in PCNL is meaningful, safe, and feasible.

## Introduction

Percutaneous nephrolithotomy (PCNL) is the standard treatment for larger (> 2 cm) renal stones [1]. The aim of every procedure should be removal of all stone material, since residual fragments can lead to progression, symptoms and the need for re-intervention [2–4]. According to the American Urological Association guideline, the overall stone-free rate for PCNL is reported to be 78% [5]. To achieve this stone-free rate, an average number of 1.9 PCNL procedures per stone episode is required. Therefore, stone-free rates after a single PCNL procedure (one-step stone-free rates) are significantly lower. Stone-free rates decrease with increasing stone size [6] and residual stones are seen more in procedures involving staghorn stones [7]. Stone-free rates in literature are generally overestimated as the majority of studies rely on X-ray or ultrasound for assessment of the stone-free status instead of computed tomography (CT) [6].

These data suggest that there is substantial room for increasing stone-free rates. An increase in one-step stone-free rates would likely result in a decrease in patient morbidity and costs for the society. This increase could be achieved by improving the intraoperative imaging modalities to visualize residual stone fragments intraoperatively. The reference imaging modality for assessment of a stone-free status is low-dose, noncontrast enhanced abdominal computed tomography (NCCT) [8]. However, these images cannot be acquired intraoperatively. Merely based on nephroscopy and fluoroscopy, it can be difficult for the urologist to conclude a stone-free status. In many cases, residual fragments are visualized on CT even though the surgeon had concluded a stone-free status at the end of the procedure [9].

An image modality that could facilitate the intraoperative assessment of the stone-free status is cone beam computed tomography (CBCT). CBCT allows for intraoperative high-resolution cross-sectional and three-dimensional imaging. In maxillofacial surgery, CBCT-imaging has been used extensively since the beginning of the twenty-first century. The CBCT-scanners used in maxillofacial surgery provide images with reduced radiation dose and superior image resolution as compared to conventional CT [10]. CBCT has also been used intraoperatively in several other fields of medicine, including neuro-endovascular surgery [11], cardiothoracic surgery [12] and orthopedic surgery [13]. With the emergence of hybrid operating rooms, CBCT is becoming more readily available. In PCNL, CBCT can be used at the end of the procedure to determine whether a stone-free status has been reached. With these images, any visualized residual fragments can still be attempted to be removed within the same procedure.

Several publications have already stated the possible benefits of CBCT for detecting residual stones in PCNL [14, 15], or have already reported on cases in which CBCT was used for this purpose [16]. However, there are not much data available about the added value of the CBCT-scanner for detecting and extracting residual fragments.

The purpose of this study was to get insight into the feasibility of using the CBCT-scanner for detecting residual stones during PCNL.

## Materials and methods

### Patient selection

This article describes a single-center observational study, conducted in a tertiary-referral hospital with an endo-urology department specialized in complex stone surgery. Between November 2018 and February 2019, all eligible patients that underwent PCNL or endoscopically combined intra-renal surgery (ECIRS) were counseled for inclusion.

This study has been approved by the local Medical Ethics Committee.

Patients below 18 years of age were excluded. Informed consent was obtained from all individual participants included in the study.

### Procedures and stone-free rates

Patients underwent the PCNL as in standard procedure. A CBCT-scan was made at the end of the procedure, where the urologist thought to have obtained a stone-free status by means of nephroscopy and C-arm fluoroscopy, and would otherwise have ended the procedure. Before making the CBCT-scan, the access sheath for the nephroscope was removed and a guide-wire was left in place. This was done to minimize scatter artifacts caused by the metal objects. If the sheath is left in place during the CBCT-scan, the scatter artifacts will lower the image quality of the CBCT-scan and thereby reduce the detectability of residual stones. A short apnea was induced by the anesthesiologist to reduce movement during image acquisition. Then, all personnel were moved to a lead glass shielded area. After interpretation, any residual fragments imaged on the CBCT-scan could then still be attempted to be extracted in a continued procedure, after re-introducing the sheath and nephroscope. A low-dose NCCT-scan was made 4 weeks postoperatively as is standard follow-up care in our center. The scans were assessed and compared by the main investigator. Two definitions of a stone-free status were used; fully stone-free with no residual fragments on follow-up CT and as is common in literature, a definition of stone-free with residual fragments ≤ 4 mm in maximum diameter.

### Cone beam CT-protocol

**Fig. 1:**
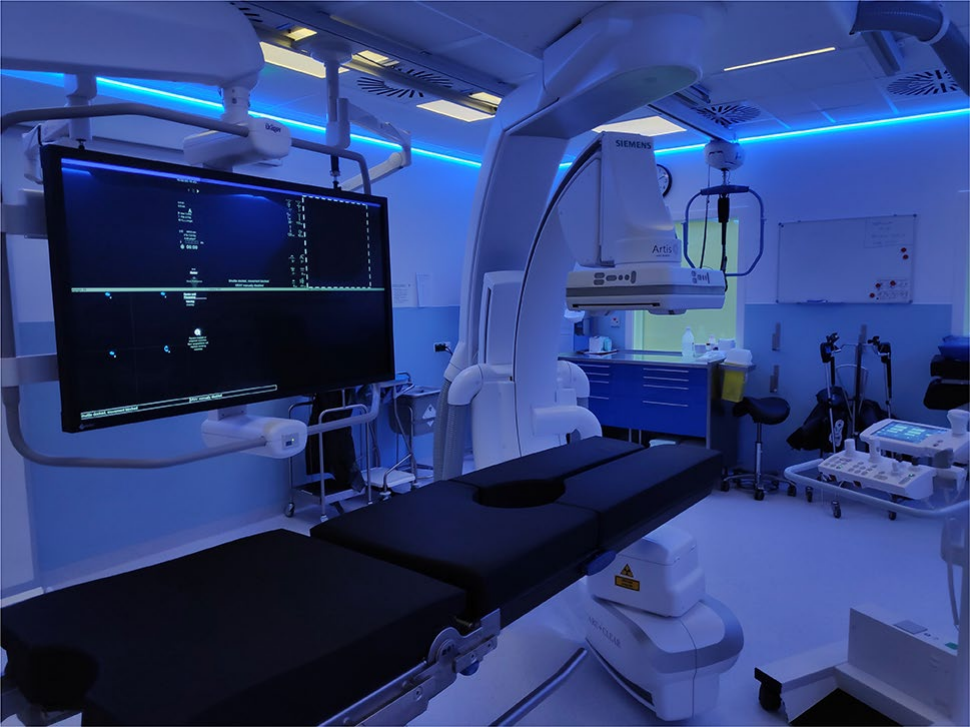
The Cone Beam CT-scanner in the hybrid operating room in the urological intervention center, University Medical Center Groningen

The CBCT-scanner used in this study is an Artis Q Ceiling DynaCT (Siemens Healthcare), installed in a hybrid operating room (Fig. 1). It combines standard fluoroscopy with CBCT-imaging. A dedicated workstation performs three-dimensional image reconstruction and image post-processing with reconstruction software. The CBCT-protocol that was used had an image acquisition time of 6 s, with a total of 397 frames and a radiation dose of 0.36 μGy/f. Three-dimensional image reconstructions are made for every CBCT-scan and are available within 5 s after image acquisition is complete.

Slice thickness can be can be altered with a slider at any moment in the viewing options of the CT-scan viewing software, with a minimal slice thickness of 0.1 mm. Default viewing slice thickness was set at 2.0 mm. Slice increment can be varied as well, with a default setting of 1 mm in this study.

### Time registration

During the procedures, several timestamps were registered at certain stages of the procedures. These stages included the start of preparing for the CBCT-scan, the end of interpretation of the CBCT-scan, the start of any additional stone treatment after the CBCT-scan and the end of this stone treatment. With these timestamps, the required time for preparing, making and interpreting the CBCT-scan could be calculated as well as the required time for additional stone treatment.

### Radiation exposure

Radiation exposure of the CBCT-scans was tracked with dose reports provided by the DynaCT software. This dose report lists a dose area product (DAP) per scan, in μGym^2^. A conversion factor of 0.13 mSv Gy^−1^ cm^−2^ was used to calculate the effective dose in mSv [17]. It must be noted that the effective dose calculated with this method is an estimate. DAP-conversion factors vary strongly among different cone beam CT-scanners and are dependent on patient BMI as well [17]. However, the conversion factor can provide a clear estimate of the mean effective dose.

### Statistics

All statistical analyses were performed using the statistical package for the social sciences (SPSS) for Windows (version 23.0).

## Results

In 19 procedures during the study period, both an intraoperative CBCT-scan and an NCCT were performed. One patient in the study population underwent a bilateral PCNL procedure with an interval of 3 weeks. Therefore, the study population consists of 18 patients, with 19 procedures to be analyzed.

### Baseline characteristics

Of the 18 study patients, 50% were male (*n* = 9) and 50% were female (*n* = 9), with a mean age of 56 years. The median BMI (body mass index) was 26.7 kg/m^2^. Out of 19 procedures, 3 cases (15.8%) involved a single stone below 20 mm on pre-operative imaging, 11 (57.9%) were performed for conventional stones (either multiple stones or a single stone with a diameter larger than 20 mm) and 5 (26.3%) procedures involved staghorn stones. These characteristics are displayed in Table 1.

**Table 1:** Baseline characteristics (*n* = 18)

Age in years	
Median (range)	60 (32–77)
Mean (SD)	56 (13.5)
Gender	
Male; *n* (%)	9 (50)
Female; *n *(%)	9 (50)
BMI; kg/m^2^	
Median (range)	24.4 (18.9–36.81)
Mean (SD)	26.7 (5.8)
Stone type	
Single stone < 20 mm; *n* (%)	3 (15.8)
Single stone > 20 mm; *n* (%)	3 (15.8)
Multiple stones; *n* (%)	8 (42.1)
Staghorn stone; *n* (%)	5 (26.3)

PCNL-monotherapy was performed in 12 (63.2%) procedures, whereas 7 (36.8%) procedures were combined-approach procedures (ECIRS). Patients were in the prone position in 12 cases (63.2%) and in the supine position in 7 cases (36.8%). Full-size PCNL (Storz MIP-L, 24F) was used in 13 cases (68.4%), mini PCNL (Storz MIP-M, 16.5F) in 4 cases (21.1%) and ultra-mini PCNL (Schölly UMP, 13F) in 2 cases (10.5%). Further procedure characteristics are displayed in Table 2.

**Table 2:** Procedure characteristics (*n* = 19)

Procedure	
PCNL-monotherapy; *n* (%)	12 (63.2)
ECIRS; *n* (%)	7 (36.8)
Side	
Left; *n* (%)	13 (68.4)
Right; *n* (%)	6 (31.6)
Position	
Prone; *n* (%)	12 (63.2)
Supine (straight legs); *n* (%)	5 (26.3)
Supine (legs in stirrups); *n* (%)	2 (10.5)
PCNL size	
Ultra-mini PCNL (13F); *n* (%)	2 (10.5)
Mini PCNL (16.5F); *n* (%)	4 (21.1)
Full-size PCNL (24F); *n* (%)	13 (68.4)
Stone treatment	
Ultrasound lithotripsy	8 (42.1)
Laser lithotripsy	5 (26.3)
Ballistic lithotripsy	2 (10.5)
Basket/forceps	2 (10.5)
Flushing	2 (10.5)

### Extraction of residual fragments

In 9 out of the 19 PCNL procedures (47%), one or more residual fragments that were visible on the CBCT-scan could still be extracted. In 6 out of 19 (26%) procedures, calcifications were imaged on the CBCT-scan but could not be retrieved. These calcifications are thought to be either stones in inaccessible calyces, parenchymal calcifications or Randall’s plaques, or residual fragments that could not be located despite thorough examination. It can be difficult to distinguish between these mentioned explanations. In one case, the procedure had to be ended due to its long duration.

### Image quality

The quality of images was compared between the intraoperative CBCT-scans and the follow-up NCCT-scans.

No follow-up NCCT-scans revealed residual fragments that were not imaged on CBCT-scans. Image quality was comparable between the two types of scans.

**Fig. 2:**
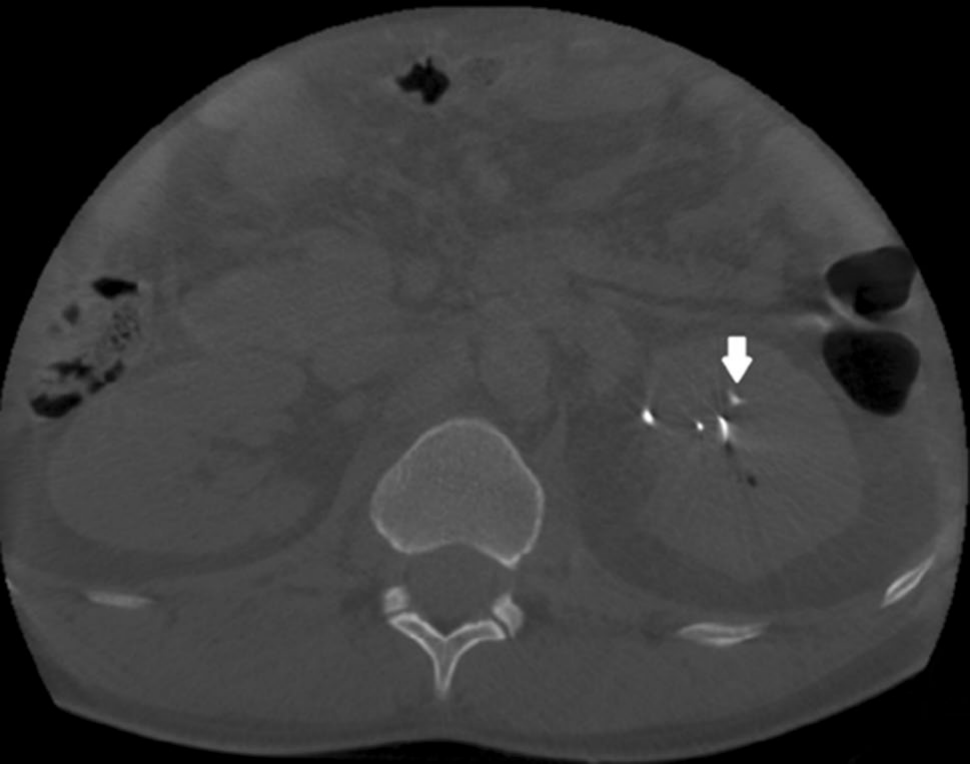
Example of an intraoperative CBCT-scan. The white arrow indicates a residual fragment that was extracted after acquiring the CBCT-images. The other white structures represent the occlusion catheter in the ureter and the safety wire

Figure 2 shows an example of a CBCT-scan image in a patient with a residual fragment which was removed intraoperatively.

**Fig. 3.**
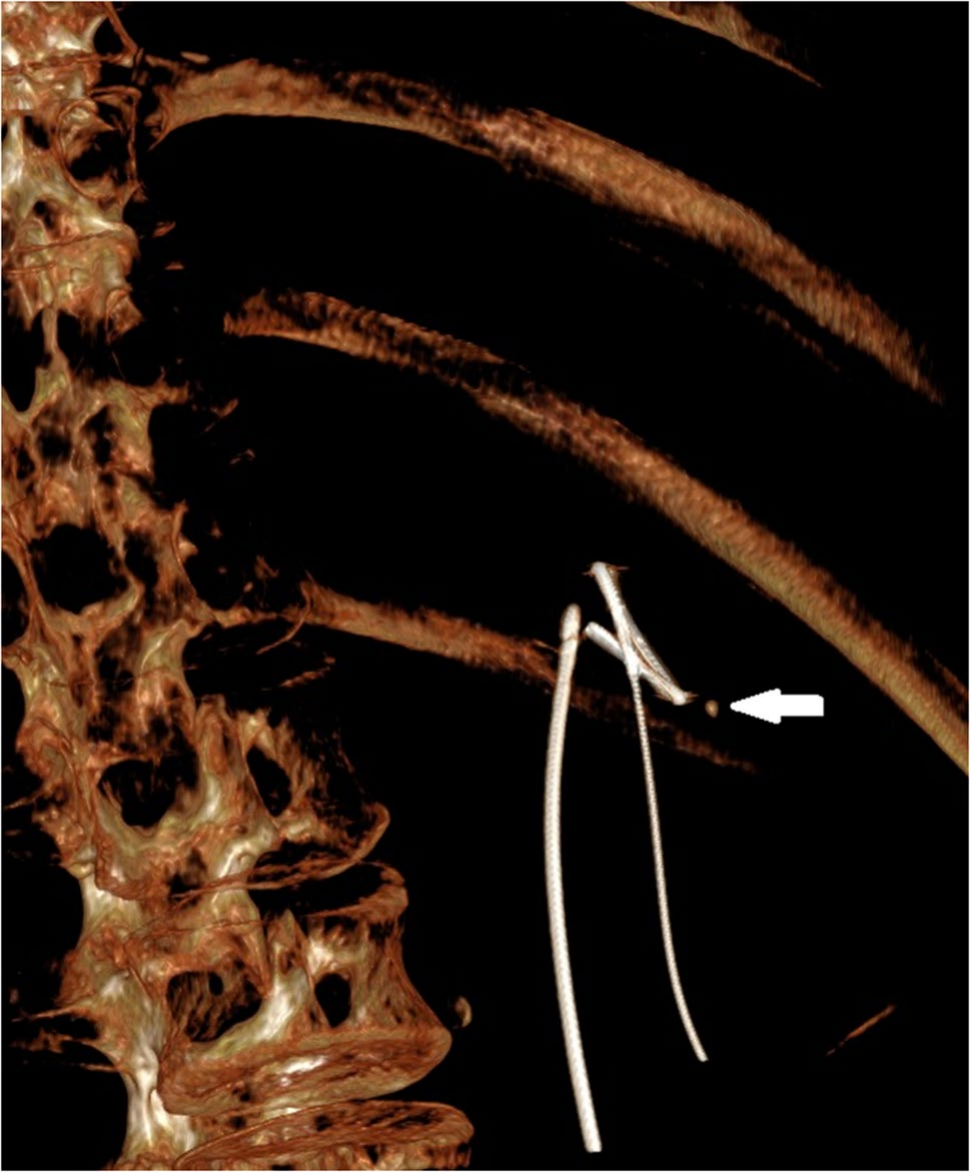
3D-reconstruction of the CBCT-scan in a patient with a residual fragment. The residual fragment is indicated by a white arrow. The straight wire represents an occlusion catheter. The wire with a loop represents the safety wire

Figure 3 shows a part of the 3D-reconstruction of the CBCT-scan for this patient.

### Stone-free status

Of the 19 procedures, 4 patients were fully stone-free on the CBCT-scan (21.1%). In 5 procedures, residual fragments below or equal to 4 mm in diameter were seen on the CBCT-scan (26.3%). In the remainder of 10 procedures, the CBCT-scan showed residual fragments above 4 mm in diameter (52.6%). Including RFs below 4 mm, this gives a stone-free rate of 47.4%. For 5 out of 19 procedures, a fully stone-free state was imaged on the follow-up NCCT-scan (26.3%). In 7 NCCT-scans, residual fragments below or equal to 4 mm in diameter were seen (36.8%). This leaves 7 NCCT-scans with residual fragments above 4 mm in diameter (36.8%). With a definition of stone-freedom including RFs below 4 mm, this results in a stone-free rate of 63.2%.

**Fig. 4:**
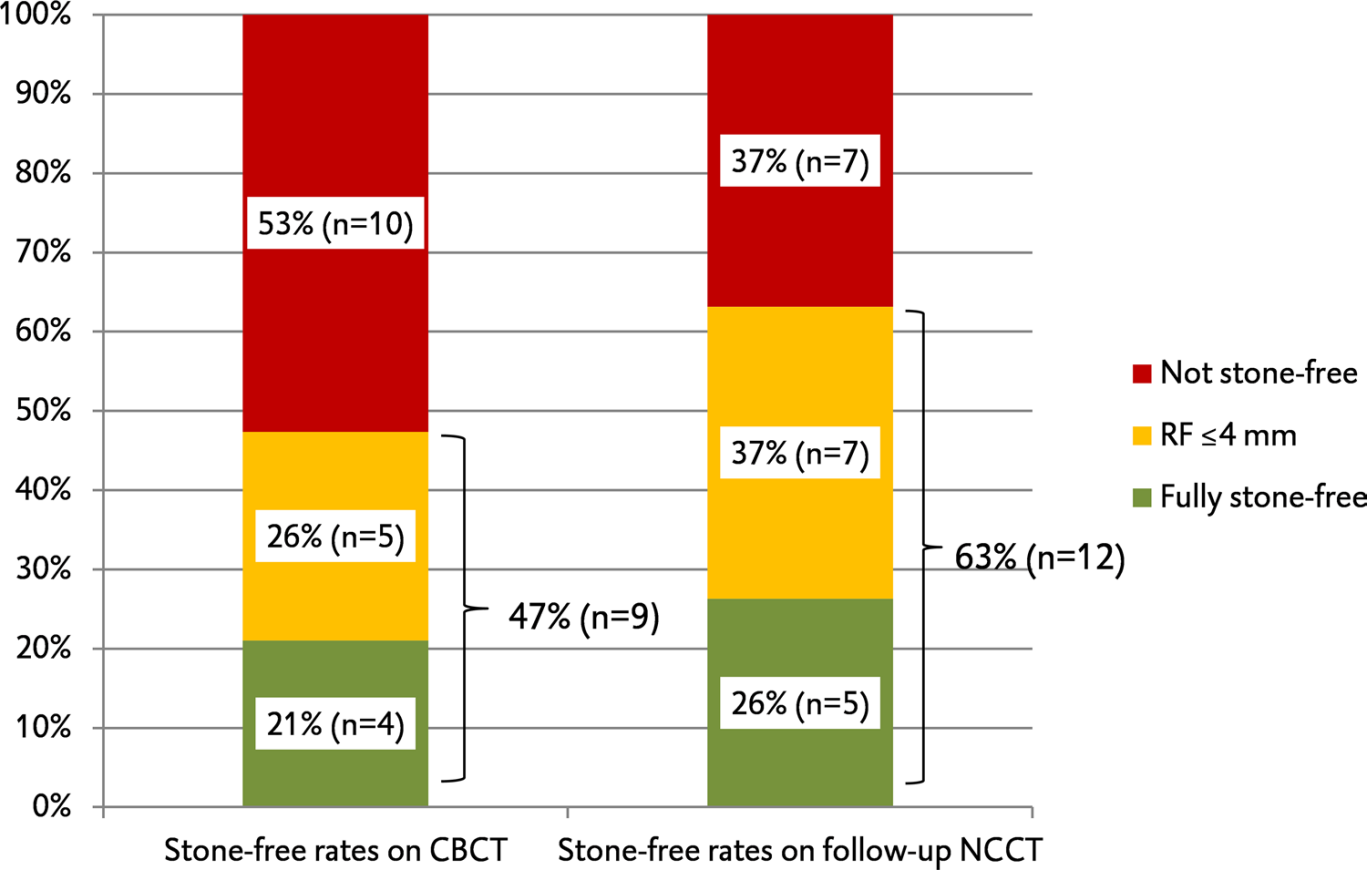
Stone-free rates of CBCT-scans and follow-up NCCT-scans

The comparison of stone-free rates is displayed in Fig. 4.

Out of the 7 cases in which ECIRS was used, intraoperative CBCT-scans showed RFs larger than 4 mm in 5 cases, and in 2 cases, RFs smaller than 4 mm. None of these 7 ECIRS cases were fully stone-free on intraoperative CBCT-scan. In 4 out of the 7 ECIRS cases, residual fragments were extracted after performing the CBCT-scan. In the 3 cases where no residual fragments could be extracted despite the use of ECIRS, the calcifications could not be located in two cases. In the remaining case, the procedure had to be ended because of its long duration.

### Duration of making CBCT-scans

The median duration of preparation, performing and interpreting the CBCT-scan was 8 min (range 4–24 min). In the procedure where this duration was 24 min, technical issues delayed making the CBCT-scan. The median duration of extraction of any found residual fragments on the CBCT-scan was 11 min (range 4–27 min).

### Radiation exposure

With the used conversion factor, the mean effective dose calculated for the cone beam CT-scans was 7.25 mSv, with a standard deviation of 2.21 and a range between 3.37 and 10.95 mSv.

## Discussion

Obtaining a stone-free status is the cornerstone of percutaneous nephrolithotomy. The importance of full clearance of stone material cannot be stressed enough. In this study, we examined the feasibility of the CBCT-scanner in detecting the residual fragments intraoperatively for the purpose of removing additional stone fragments.

In 9 out of 19 procedures (47%), one or more residual fragments that were visible on the CBCT-scanner were eventually removed. In these cases, the procedure would otherwise have been terminated with a higher chance of recurrence of disease symptoms. This shows that in many cases, stone fragments can still be found and extracted even when the case was decided to be clinical stone-free by means of nephroscopy and fluoroscopy.

The stone-free rate on the follow-up scans was 15.8 percent higher than that of the CBCT-scans. If this effect would be the true difference in stone-free rate for procedures with versus without a CBCT-scanner, this would be highly clinically relevant. One could argue that the difference in the calculated stone-free rates could be partially explained by the effect of spontaneous passage in the 4 weeks between the intervention and the follow-up scan. However, in all cases that transferred from not stone-free on the CBCT-scan to stone-free on the follow-up CT-scan, residual fragments of substantial size were removed after making the CBCT-scan. There were no cases that were stone-free on follow-up but not-stone-free on the CBCT-scan, where no residual fragments were removed. This supports the idea that the difference in stone-freedom is for an important part caused by the extraction of found residual stones. Furthermore, this also stresses the need for complete removal of stone material, since this suggests that the rate of spontaneous passage does not seem to be high in this study.

The high degree of imaged residual fragments on the intraoperative CBCT-scans in the patients that underwent ECIRS can be explained by the complexity of the ECIRS cases. All ECIRS cases involved either large stones, multiple stones or staghorn stones. In the many of these cases, prior stone treatment by means of either extracorporeal shock-wave lithotripsy, ureterorenoscopy or PCNL had failed.

The added median surgery duration of 8 min for performing and interpreting the CBCT-scan and 11 min for extracting any imaged residual fragments seem acceptable if this could delay recurrent stone disease with possible additional morbidity.

According to the guidelines on diagnosis of urolithiasis of the European Urological Association (EAU) [18], low-dose NCCT results in an effective dose of 0.97–1.9 mSv, regular dose NCCT leads to 4.5–5 mSv and enhanced CT has an effective dose of 25–35 mSv. The dose of the cone beam CT-scans in our study had a mean calculated effective dose of 7.25 mSv, which is higher than the dose of a regular NCCT, but far lower than enhanced CT. Radiation dose could be lowered by implementing low-dose CBCT-protocols, as described by Rassweiler et al. [19]. Future research is needed to find the optimal scanning parameters to balance between radiation dose and stone detectability.

Stone-free rates observed in this study were relatively low as compared to stone-free rates in literature. This can be explained by the high degree of complex stone cases in our tertiary care hospital. Furthermore, our study describes one-step stone-free rates whereas many other studies describe a stone-free rate of a stone episode with several procedures.

Another factor accounting for the relatively low stone-free rates in this study, is the imaging modality. Where most studies rely on X-ray or ultrasound imaging for assessment of the stone-free status, CT-scans provide the highest sensitivity and specificity for stone detection [20].

### Study limitations

A factor that limits the value of the data in this study is a possible investigator-induced bias. In this study, a situation without the CBCT-scanner is simulated, using the CBCT-scan at the moment where the procedure would otherwise be ended. Since it is known that the CBCT-scan will be made in all cases, the outcomes of the CBCT-scans could be influenced. A study design that would eliminate this effect is a randomized controlled trial, where patients are randomized into CBCT and non-CBCT arms, and the allocation of the subjects is only revealed to the urologist at the moment when the CBCT-scan is requested to be made.

The effect of spontaneous passage between the end of the procedure and the follow-up NCCT-scan is not known. Though this effect seems small as mentioned above, the observed difference in stone-free rates is influenced to an unknown extent.

Another significant limitation in this study is the small sample size, which makes it practically impossible to show statistically significant effects in this study. We estimate that the measured stone-free rate in this feasibility study is lower than the overall stone-free rate in a larger study population would be, since the stone load and surgical difficulty of the cases in this study were challenging. Further research is needed to assess the efficacy of using the CBCT-scanner in increasing stone-free rates.

## Conclusion

The intraoperative use of a CBCT during PCNL to visualize residual fragments which are not detected by means of the conventional techniques is a safe and feasible strategy. A number of additional stones were removed guided by the CBCT and we demonstrated an increased stone-free rate at follow-up CT. Whether this results in a relevant increase in one-step stone-free patients remains unclear because the impact of spontaneous stone passage is unknown. To further examine the advantages of CBCT, a randomized controlled trial will be performed to minimize the mentioned study limitations.

## Abbreviations

BMI: Body mass index
CBCT: Cone beam computed tomography
CT: Computed tomography
ECIRS: Endoscopically combined intra-renal surgery
NCCT: Noncontrast enhanced abdominal computed tomography
PCNL: Percutaneous nephrolithotomy

## Declarations

### Availability of data and material

All data are stored in a protected database on a server of the University Medical Center Groningen.
